# The Effect of Human Papillomavirus Infection on Pregnancy Outcomes: A Scoping Review

**DOI:** 10.3390/diagnostics16040629

**Published:** 2026-02-21

**Authors:** Borek Sehnal, Jan Zapletal, Martin Hruda, Vit Drochytek, Katerina Maxova, Michael J. Halaska, Lukas Rob, Ruth Tachezy

**Affiliations:** 1 Department of Obstetrics and Gynaecology, University Hospital Kralovske Vinohrady and Third Faculty of Medicine, Charles University, 100 34 Prague, Czech Republic; jan.zapletal@fnkv.cz (J.Z.); martin.hruda@fnkv.cz (M.H.); vit.drochytek@fnkv.cz (V.D.); katerina.maxova@fnkv.cz (K.M.); michael.halaska@fnkv.cz (M.J.H.); lukas.rob@fnkv.cz (L.R.); 2 Department of Genetics and Microbiology, Faculty of Science, Biotechnology and Biomedicine Centre of the Academy of Sciences and Charles University, Charles University, 128 00 Prague, Czech Republic

**Keywords:** human papillomavirus, pregnancy, adverse pregnancy outcomes, scoping review, miscarriage, preterm birth, premature preterm rupture of membranes, preeclampsia, fetal growth restriction, intrauterine fetal death

## Abstract

**Background:** Human papillomavirus (HPV) is the most common sexually transmitted viral infection worldwide. Moreover, the prevalence of HPV infection is twice as high in pregnant women as in non-pregnant individuals. The aim of this review was to examine adverse pregnancy outcomes associated with cervicovaginal or placental HPV infection confirmed by a sensitive molecular method. **Methods:** We conducted searches on major medical databases including PubMed, EMBASE, Global Health, and the Cochrane Library to identify all studies examining HPV infection during pregnancy. Additionally, other online sources were consulted for relevant studies. Thirty-four records out of the initial 1868 identified were included in this review for thematic synthesis. The PRISMA-ScR guidelines were followed. **Results:** This scoping review included a total of 28 original observational studies, 1 systematic review, and 5 meta-analyses. Active HPV infection appears to significantly increase the risk of preterm premature rupture of membranes and preterm birth, as indicated by findings from published meta-analyses and systematic reviews. Determining the association of HPV infection with certain adverse pregnancy outcomes is challenging due to their frequency (such as miscarriage) or rarity (such as intrauterine fetal death). For conditions like preeclampsia and intrauterine fetal growth restriction, the limited number of heterogeneous studies precludes definitive conclusions. Moreover, the causes of these outcomes are typically multifactorial. The presence of HPV in trophoblasts and placental tissue is considered crucial for potential adverse pregnancy outcomes. There appears to be a strong correlation between cervicovaginal or urinary HPV infections and placental HPV infections in pregnant women. **Conclusions:** Persistent HPV infection seems to elevate the risk of preterm premature rupture of membranes and preterm birth. However, the currently available observational evidence does not allow for definitive conclusions regarding causality, and the reported findings should be interpreted as associations rather than proof of a causal relationship. The changes in frequency of certain perinatal complications in populations of women with high HPV vaccination rates may shed more light on this connection.

## 1. Introduction

The human papillomavirus (HPV) is a group of viruses that 75–80% of sexually active individuals are infected with at least once during their lifetime. It is therefore the most common sexually transmitted viral infection worldwide [1]. HPV viruses infect the skin and mucous membranes of the anogenital and orofacial areas. Infection is often asymptomatic and resolves spontaneously over time. However, in a small percentage of infected individuals, the virus persists with an increased risk of tumour transformation of the infected tissue. The DNA of some genotypes integrates into the genome of the host infected cell with subsequent increased activation of expression of viral oncogenes E6 and E7, which can lead to malignant transformation of the cell. However, there are other mechanisms by which HPV without integration into the host genome increases the risk of malignancy. Based on the ability of each genotype to initiate carcinogenesis, HPV is divided into high-risk (HR) and low-risk (LR) [2]. HPV is the most common infectious cause of malignancy [1,3]. According to the International Agency for Research on Cancer (IARC), 13 genotypes are currently classified as HR HPV, with HPV genotypes 16, 18, 31, 33 and 45 considered the most important for the development of human malignancies [2]. The total number of HPV-related cancers has been estimated to account for approximately 4.5% of all human cancers worldwide each year, including 8.6% in women and 0.8% in men [4].

Although the greatest risk of HPV infection lies in the initiation of benign and malignant tumours, HPV infection can also adversely affect the course of pregnancy. Most published studies have found a higher prevalence of HPV infection in pregnant women compared to non-pregnant women. A recent 2023 meta-analysis of 144 studies reported a global prevalence of HPV in pregnancy of up to 30.4% (95% CI: 26.9–34.0) in the cervix and vagina, 17.8% (95% CI: 9.8–27.5) in the placenta, 32.1% (95% CI: 25.1–39.7) in serum, 2.3% (95% CI: 0.1–8.1) in amniotic fluid, and 25.5% (95% CI: 23.3–27.8) in urine and the urinary tract. The highest prevalence was observed in populations in Africa and the lowest in Europe and the Eastern Mediterranean [5]. The exact consequences and implications of HPV infection in pregnant women have not yet been studied in detail, but there is evidence for mechanisms of adverse effects of HPV infection in pregnancy. The aim of this scoping review article is to summarize current knowledge of the impact of active HPV infection on pregnant women and unborn children.

## 2. Materials and Methods

### 2.1. Design

A scoping review framework was selected to guide decision-making by identifying and analyzing the literature on a specific topic [6]. This review adhered to the following steps: identifying the research question, locating relevant studies, selecting studies, charting the data, summarizing and reporting the findings, and consulting with knowledge users [7]. Ethical approval was not required since this review methodology did not involve experimental research or human participants.

### 2.2. Population of Interest

We included studies investigating adverse pregnancy outcomes in women with and without HPV infection in various genital sites (vulva, vagina, cervix), placenta, or other products of conception. HPV status was required to be determined using a sensitive molecular method, namely PCR (polymerase chain reaction) or HCA (hybrid capture assay). Studies reporting both general HPV infections and type-specific infections were included.

Two main research questions guided the scoping review:

1. Is there a significant association between HPV infection, as detected by PCR or HCA in the lower genital tract or trophoblastic tissue (placenta), and adverse pregnancy outcomes?
2. Can we estimate the effect of HPV infection on the risk of miscarriage (spontaneous abortion), preterm birth, premature preterm rupture of membranes (PPROM), preeclampsia, fetal growth restriction (FGR), and intrauterine fetal death?

Additionally, we aimed to determine the strength of the association between cervicovaginal or urinary HPV infection and the presence of HPV infection in trophoblasts.

### 2.3. Search Strategy

The following databases were searched to identify all suitable studies: Medline PubMed, EMBASE, Global Health, and the Cochrane Library (Table 1, Search strategy). A sensitive literature search strategy was developed to locate published peer-reviewed literature, with search terms combined using Boolean operators. The terms used in various combinations included the following: (“human papillomavirus” OR “HPV”) AND (“pregnancy” OR “miscarriage” OR “spontaneous abortion” OR “pregnancy loss” OR “preterm birth” OR “premature rupture of membranes” OR “PROM” OR “preeclampsia” OR “eclampsia” OR “pregnancy-induced hypertension” OR “PIHD” OR “fetal growth restriction” OR “FGR” OR “intrauterine growth restriction” OR “IURG” OR “small for gestational age” OR “SGA” OR “intrauterine fetal death” OR “stillbirth” OR “adverse birth outcome”). The search was limited to research published in English from 1 January 2000 to 31 August 2025.

We conducted a scoping review following the standard six-stage approach [8] and adhered to the Preferred Reporting Items for Systematic Reviews and Meta-Analyses (PRISMA) guidelines and its extension for scoping reviews [9]. We did not use any registered review protocol.

### 2.4. Types of Studies

We included all types of observational studies: cohort (both retrospective and prospective), case–control, and cross-sectional studies. Additionally, we considered data from systematic reviews and meta-analyses.

### 2.5. Inclusion and Exclusion Criteria

Full-text evaluation was conducted based on the inclusion and exclusion criteria and the availability of the necessary data to address the main question of this review. Any disagreements were resolved through discussion in an expert-led environment. Due to the specific focus of this review and the publication repertoire in online databases, we did not explicitly search for additional grey literature.

### 2.6. Data Extraction

Data extraction was initially performed by one reviewer using a predefined data extraction framework, with three authors determining the relevant variables to be collected from the included studies. To ensure accuracy and minimize potential bias, the extracted data were subsequently reviewed and cross-checked by additional members of the research team. Key study characteristics and outcome measures were independently verified, and any discrepancies were resolved through discussion and consensus within the author group. This iterative process was conducted throughout the review to enhance methodological rigour and data reliability (Figure S1, engagement strategy framework). Information extracted from each study included publication characteristics, study design parameters, and pregnancy outcomes among HPV-positive individuals, following the PRISMA Extension for Scoping Reviews (ScR) 2020 guidelines [9].

## 3. Results

**Figure 1:**
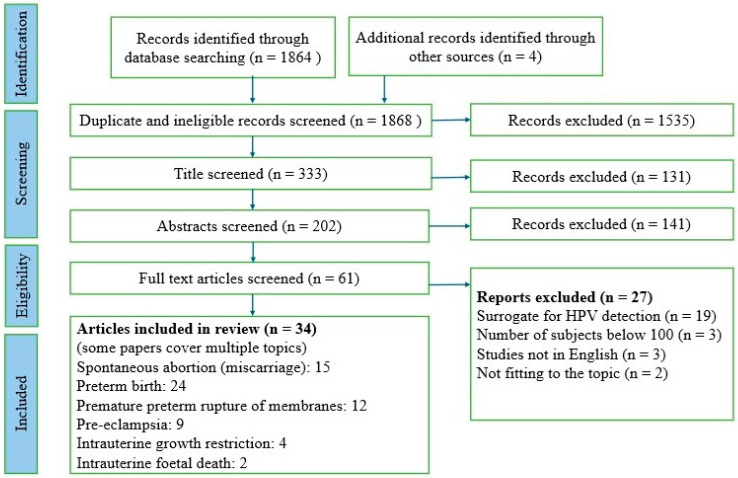
PRISMA 2020 flowchart representing the study selection process.

A total of 1864 titles from the main databases and 4 titles from other databases were identified and verified (Figure 1, PRISMA 2020 flowchart representing the study selection process) [10]. Out of these, 1535 articles were either duplicates or ineligible for other reasons and were excluded. Another 131 articles were excluded after screening titles, and 141 studies were excluded after reviewing abstracts for not fitting our topic and inclusion criteria. No additional articles were found during the reference list screening.

Finally, 61 articles were screened by reading the full text, resulting in the inclusion of 28 original observational studies [11,12,13,14,15,16,17,18,19,20,21,22,23,24,25,26,27,28,29,30,31,32,33,34,35,36,37,38], one systematic review [39], and five meta-analyses [40,41,42,43,44]. Review articles published without clear statistical data were not included [45,46,47,48,49,50]. The final search results were exported into the Zotero program. The search strategy and inclusion criteria were verified by three reviewers. The characteristics and results of the included studies are presented in Table 2 (characteristics of included studies), while the summary results of meta-analyses on the effect of HPV on the course of pregnancy are shown in Table 3 (results of published meta-analyses).

### 3.1. Miscarriage (Spontaneous Abortion)

Miscarriage (spontaneous abortion) and stillbirth are two general terms describing the death of the fetus, but they refer to losses that occur at different times during pregnancy. The various definitions used therefore pose a methodological difficulty when attempting to interpret and accurately compare stillbirth rates and associated risk factors [51]. WHO recommends a weight of 500 g as the cut-off point between miscarriage and stillbirth [52]. More than half of miscarriages are thought to be due to chromosomal abnormalities in the first trimester of pregnancy [53]. Other common causes include maternal or fetal infections.

The scoping review identified 11 studies [12,13,14,15,18,21,23,28,29,31,38], 1 systematic review [39], and 3 meta-analyses [41,43,44]. The outcomes of the individual studies were inconclusive. For example, in his systematic review, Ambühl pointed to a higher prevalence of HPV in women who had spontaneous abortion but without clear evidence of HPV infection as the cause. HPV prevalence in women with spontaneous abortion was statistically significantly different from women with term delivery in the cervix only (24.5% vs. 17.5% *p* < 0.05) and in the placenta (24.9% vs. 8.3%; *p* < 0.05) [39]. These results are consistent with those of some earlier studies. Bennani et al. demonstrated HPV infection as a risk factor for spontaneous abortion (aOR 3.76, *p* = 0.001) [13]. Ticconi et al. detected a lower prevalence of HPV DNA on the cervix in patients with recurrent unexplained pregnancy loss (26.5%; 13/49) compared to women who had delivered at least one fetus at term and no history of miscarriage (61.9%; 294/475; *p* < 0.001). The authors hypothesized that the increased immune response of the body potentially responsible for recurrent spontaneous abortion is also a protective factor against HPV infection [31]. Similarly, Bober et al. observed a statistically significant higher prevalence of HPV infection in cervical smears and trophoblasts in women with abnormal first trimester pregnancy [14]. HPV was detected in 17.9% (15/84) of patients in the study group and in 6.8% (4/59) of patients in the control group.

Similarly, HR HPV infection was significantly more common in patients with abnormal first-trimester pregnancy (15.5% vs. 5.1%; *p* = 0.03). Moreover, HR HPV trophoblast infection was found only in patients in the study group (9.5% vs. 0.0%; *p* = 0.02) [14]. While these three studies [13,14,31] and a systematic review [39] documented HPV infection as a significant risk factor for miscarriage, the other seven studies [12,15,18,21,23,28,29] indicated a potential risk of HPV infection but without statistical significance (Table 2, characteristics of included studies). A study of 281 women in Mexico found a prevalence of cervical HPV infection in 24.4% of women with first-trimester spontaneous abortion and 15.2% of controls, with HPV 16 and HPV 58 genotypes being the most detected in both groups. Anamnestically, 27.3% of HPV-positive and 17.4% of HPV-negative women reported at least one previous pregnancy loss; however, HPV was not statistically significantly associated with a single or recurrent spontaneous abortion. A history of spontaneous abortion was significantly more frequently associated with age over 35 years, alcohol consumption and multiple sexual partners [18]. In a study from Poland, HPV DNA was detected in chorionic tissue in 17.7% (9/51) of women with spontaneous abortion between 6 and 16 weeks of pregnancy and in 24.4% (19/78) of placentas of women who delivered at term (*p* = 0.366). The prevalence of HPV 16/18 detection was not statistically different between the cohorts (11.8% vs. 12.8%; *p* = 0.859) [29].

The 2018 meta-analysis included 12 studies on a total of 3007 patients with different outcomes. Five cohort and three case–control studies found a higher risk of spontaneous abortion associated with HPV infection (OR 1.40; 95% CI: 0.56–3.50), with the cohort studies having a higher risk (OR 1.47; 95% CI: 0.86–2.50) than the case–control studies (OR 1.02; 95% CI: 0.21–5.02). However, the presence of HR HPV infection alone (four studies) did not support an increased risk of miscarriage (OR 0.65; 95% CI: 0.21–1.98). Conversely, the other four studies calculated more than twice the odds of spontaneous miscarriage when any HPV infection was detected (OR 2.24; 95% CI: 1.37–3.65) [43].

The evidence regarding the association between HPV infection and miscarriage remains inconsistent, and the observed findings appear to be largely influenced by methodological differences across individual studies. While some investigations have demonstrated a statistically significant increased risk of spontaneous abortion among HPV-positive women, others have found no association. Variability in the published results is largely attributable to differences in study design, sample size, and, importantly, the anatomical site of HPV detection. Some studies assessed cervical HPV infection, whereas others examined trophoblastic or placental tissue. Cervical HPV detection may reflect transient mucosal colonization rather than direct placental involvement; thus, studies relying exclusively on cervical samples may either overestimate or underestimate the biological relevance of HPV infection for early pregnancy loss. In contrast, studies detecting HPV DNA in trophoblastic tissue may better represent a direct mechanistic association, although they are often limited by smaller sample sizes.

Adjustment for potential confounders further contributes to heterogeneity. Maternal age, smoking status, previous obstetric history, sexual behaviour, and coexisting infections are established risk factors for miscarriage. Not all included studies accounted for these variables, which may partially explain the divergent findings.

Meta-analyses have also yielded inconsistent conclusions. Although some suggested increased odds of miscarriage among HPV-positive women, statistically significant associations were not consistently demonstrated.

Taken together, the current body of evidence does not allow for a definitive conclusion regarding a causal role of HPV infection in miscarriage. The observed discrepancies likely reflect methodological heterogeneity rather than a true biological contradiction. Because spontaneous abortion is the most common adverse outcome of pregnancy, it is important to further study the issue of HPV infection as a causative agent in more detail to reach more definitive conclusions [46,47,48,49].

### 3.2. Preterm Birth and Premature Preterm Rupture of Membranes (PPROM)

Preterm birth is defined as a birth that occurs before 37 completed gestational weeks (less than 259 days) of gestation. The number of preterm births has been steadily increasing worldwide over the past two decades. Prematurity is associated with a considerable risk of morbidity and mortality, particularly among extremely preterm infants (i.e., <28 weeks). Worldwide, the incidence of preterm births is estimated to be approximately 10% (range 5% in parts of Europe to 18% in parts of Africa), and approximately 15 million children are born preterm each year (range 12 to 18 million) [54,55]. In the United States, approximately 550,000 preterm infants are born each year, with about 10 percent of all live births born before 37 weeks GA and almost 3 percent born <34 weeks [56].

Preterm birth is the result of the activation of the same mechanisms as term birth, but in the case of preterm birth, their activation is pathological. Often the exact cause of preterm birth cannot be determined; the provoking factor is often an infection of the feto-maternal unit [57,58,59]. Premature rupture of membranes (PROM) is defined as the rupture of the amniotic sac (amnion) before the onset of uterine activity. If spontaneous amniotic fluid outflow occurs before 37 weeks of gestation, it is a premature preterm rupture of the membranes (PPROM); spontaneous amniotic fluid outflow before the onset of regular uterine activity at term is termed a term PROM [60].

A total of 18 studies [11,12,16,17,19,22,23,24,25,26,27,28,30,32,33,34,35,38], one systematic review [39] and five meta-analyses [40,41,42,43,44] met the inclusion criteria for the scoping review regarding preterm birth, and a total of nine studies [16,17,20,22,23,28,33,34,36] and three meta-analyses [41,42,44] were included regarding PPROM (premature preterm rupture of the membranes). Altogether, 10 studies [19,24,25,26,27,30,32,34,35,38] and a systematic review [39] demonstrated a statistically significant effect of HPV infection on the risk of preterm birth and eight studies [11,12,16,17,22,23,28,33] did not, but three of these studies [16,17,28] showed a significant risk of HPV infection for PPROM. Cotton-Caballero et al. found that preterm birth in HPV infection is often due to PPROM [61]. The 2021 HERITAGE cohort study of 899 pregnant women confirmed a nearly 4-fold increased risk of preterm birth (adjusted odds ratio, aOR 3.72; 95% CI: 1.47–9.39) with persistent vaginal HPV infection with 16/18 genotypes. Surprisingly, any vaginal HPV infection did not show a statistically significant effect (aOR 1.39; 95% CI: 0.79–2.46) [27]. The presence of placental HPV 16/18 infection was also significantly more common in women with preterm delivery (aOR 2.92; 95% CI: 1.09–7.81) [27]. Similarly, in a systematic review, Ambühl published a higher detection of HPV in the cervix (47.0% vs. 17.5%) and placenta (50.0% vs. 8.3%; *p* < 0.0001) in women with preterm birth [39]. McDonnold from the USA reports an almost 2-fold increased risk of preterm delivery before 37 weeks in the presence of cervicovaginal HR HPV infection (OR 1.83; 95% CI: 1.03–3.26) and even a 7-fold increased risk of delivery before 35 weeks (OR 6.85; 95% CI 1.87–25.09) [25]. Mosbah et al. detected HPV placental infection in 18.9% (10/53) of Egyptian women who delivered preterm and 4.0% (2/50) of women who delivered at term (*p* = 0.019). The authors suggest that HPV infection (and particularly HR genotypes) is a risk factor for preterm birth in the Egyptian population [26]. Several studies have analyzed the effect of HPV infection on PPROM. Cho et al. found that 27.3% of women with PPROM had cervical HR HPV infection, whereas in the HR HPV-negative group, PPROM was seen in only 14.2% of women (*p* = 0.029). The authors conclude that the presence of HR HPV infection is a significant risk factor for PPROM [17]. In a relatively small sample, Pandey found HPV positivity in the vagina in 14.6% of women with PPROM compared to 3.2% in the control group (*p* = 0.026) [28]. In another retrospective study of 2153 women, the presence of HPV infection approximately doubled the risk of PPROM (OR 2.07, 95% CI: 1.03–4.14) [61].

However, other studies have not produced such convincing results. Data from the Scottish registry (386 women with preterm delivery and 4942 women with term delivery) showed only a risk of preterm delivery in women with a history of surgically treated cervical precancer (OR 1.84; 95% CI 1.10–3.08, *p* = 0.020) and not with proven cervical HR HPV infection [11]. A prospective multicentre cohort study also found similar results.

HPV infection during pregnancy was not significantly associated with increased risk for the risk of spontaneous preterm birth (aOR 2.26; CI95%: 0.79–6.50, *p* = 0.13) and for PPROM (aOR 0.62; CI95%: 0.07–5.59, *p* = 0.67) [33].

However, meta-analyses (Table 3, results of published meta-analyses) and large cohort studies [34,36,38] have demonstrated quite convincingly the negative impact of HPV infection on the risk of PPROM and preterm birth. A 2014 meta-analysis of eight studies calculated the risk of preterm birth to be approximately twice as high in HPV-positive pregnant women compared to HPV-negative women (OR 2.12; 95% CI: 1.51–2.98; *p* < 0.001) [40]. Another 2018 meta-analysis (four studies, 1408 pregnant women) defined HR HPV infection as a risk factor for preterm birth (OR 2.84; 95% CI: 1.95–4.14), which was confirmed by a sub-analysis of three cohort studies (1348 pregnant women), albeit with a slightly lower risk (RR 2.37; 95% CI: 1.68–3.35) [43]. In a meta-analysis of 36 studies, Niyibizi and colleagues calculated the presence of HPV infection as a risk factor for preterm birth (aOR 1.50; 95% CI: 1.19–1.88), PPROM (aOR 1.96; 95% CI: 1.11–3.45) and TPROM (term premature rupture of membranes before the onset of uterine activity, aOR 1.42; 95% CI: 1.08–1.86) [41]. Similarly, according to a 2021 meta-analysis, HPV infection is a significant risk factor for preterm birth (OR 1.81; 95% CI: 1.25–2.62; *p* = 0.002) and also for PPROM (OR 1.74; 95% CI: 1.45–2.10; *p* < 0.00001), although, according to the authors, the reliability of the results may have been affected by the different designs in the individual studies [42]. And the most recent meta-analysis identified HPV infection also as a statistically significant risk factor for preterm birth (OR = 1.94; 95% CI: 1.31–2.87; *p* = 0.005) [44]. A large retrospective analysis of 400,583 nulliparous women from the Swedish population-based registry was published in 2021 [34]. The presence of HPV infection (detected by PCR or cervical cytology) was defined as a risk factor for preterm birth (aOR 1.19; 95% CI: 1.01–1.42; *p* = 0.042), PPROM (aOR 1.52; 95% CI: 1.18–1.96; *p* < 0.001) and TPROM (aOR 1.24; 95% CI: 1.08–1.42; *p* = 0.002), as well as for neonatal mortality (aOR 2.69; 95% CI: 1.25–5.78; *p* = 0.011). Similar results were also found for women at risk with a history of cervical surgery for precancer; only the risk of preterm delivery was higher in these women (aOR 1.85; 95% CI: 1.76–1.95; *p* < 0.001) [34].

### 3.3. Preeclampsia

Preeclampsia is a pregnancy-specific multi-organ disease of unclear etiology that is conditioned by abnormal placentation and endothelial dysfunction and is accompanied by a systemic inflammatory response. Only the presence of the placenta is required for the development of the disease without the presence of the fetus; it is, in a narrower sense, a vasculopathy with insufficient trophoblast invasion of the decidua with subsequent development of placental ischemia. The prevalence is around 4.5% of pregnant women worldwide [62,63]. The etiology of preeclampsia is multifactorial, and many risk factors have been defined [64]. Some studies point to inflammation as a trigger of endothelial dysfunction [65]. It is speculated that viral and bacterial infections, e.g., cytomegalovirus, Chlamydia pneumoniae, Helicobacter pylori and potentially human papillomavirus, may play a role in the etiopathogenesis of preeclampsia [18,47,66,67].

The scoping review included eight studies [17,19,22,23,25,28,30,37] and one meta-analysis [41]. Only two studies observed a significant effect of HPV infection on the risk of preeclampsia [25,30]. A retrospective study of 942 pregnant women concluded that HR HPV cervical infection doubles the risk of preeclampsia (10.2% vs. 4.9%; *p* = 0.004; aOR 2.18; 95% CI: 1.31–3.65) [25]. Slatter et al. found by analysis of HR HPV detection from a total of 339 placentas that the presence of HR HPV infection was significantly higher in parturients with preeclampsia, preterm delivery and delivery of a hypotrophic fetus (always *p* < 0.05) [30]. Another small study from New Zealand found a high 81% expression of E6/E7 HR HPV RNA (an indicator of active viral infection) in placentas of women with preeclampsia positive for HR HPV DNA, in contrast to 13% E6/E7 HR HPV RNA expression in placentas of women without preeclampsia (*p* = 0.0006) The authors speculate that HPV is active in the trophoblast in these women from early in placental development in the first trimester, when placental quality is determined, and thus HPV infection contributes to the increased risk of developing preeclampsia [68]. However, none of the other studies (Table 2, characteristics of included studies) included in the review nor the meta-analysis (Table 3, results of published meta-analyses) showed an effect of HPV infection on the risk of preeclampsia. Current knowledge does not allow us to establish HPV infection as a risk factor for preeclampsia or other hypertensive disorders induced by pregnancy.

### 3.4. Fetal Growth Restriction

Fetal growth restriction (FGR), formerly called intrauterine growth restriction (IUGR), is a condition in which the fetus grows slowly due to a pathological process and is unable to reach its genetically determined size. Retrospective data show that 5–10% of all pregnancies are affected by pathologically abnormal fetal growth [69,70,71]. The causes can be diverse; most often FGR develops as a result of placental pathology, e.g., in preeclampsia. Compared to normal-growing individuals, babies with FGR have a 10 times higher risk of perinatal mortality. There is no causal treatment; we try to eliminate risk factors and correct timing of delivery. The infectious etiology in the development of FGR is established for infections of the TORCH group (toxoplasma gondii, rubella, cytomegalovirus, herpes simplex viruses type 1 and 2) [72,73,74,75,76]. Some studies have also found an increased risk of FGR in association with infection with varicella-zoster virus, Treponema pallidum, Plasmodium falciparum, parvovirus B19 and also HPV [47,66,74,77,78].

Only six studies [22,23,28,36,37,38] and one meta-analysis [41] met the inclusion criteria for the scoping review. Kaur et al. analyzed the results of self-reported HPV infection from the Pregnancy Risk Monitoring System from 2004 to 2011 (26,085 subjects). HPV infection was significantly associated with low birth weight (OR: 1.94, 95% CI: 1.14–3.30), but not with preterm birth, PPROM, and preeclampsia [22]. Pandey et al. did not demonstrate the effect of HPV infection on FGR on 104 pregnant women with vaginal HPV test performed in the first trimester (4.8% vs. 4.7%, *p* = 0.100) [28]. The retrospective observational cohort study on 6285 pregnant women from Beijing did not observe HR HPV infection as a risk factor for FGR (1.8% for HR-HPV positive, 0.6% for HPV negative, *p* = 0.615) [23]. Slatter et al. collected HPV from the decidua of 339 women. HPV was more frequently detected in pregnant women with acute chorioamnionitis, FGR, and preterm delivery. A total of 76.4% (55/72) of decidua HPV-positive infants had a weight below the 5th percentile [30]. Retrospective study based on abnormal cervical cytology smear detected a 90% (95% CI: 40–150%) higher risk of giving birth to a baby weighing below the third percentile. However, this risk was only 50% (95% CI: 10–100%) when other risk factors (maternal age, ethnicity, social status, occupation, smoking, comorbidities) were eliminated [79].

HPV exposure was associated with FGR in the meta-analysis of seven studies (aOR, 1.17; 95% CI:1.01–1.37; I^2^ = 0%). Moreover, HPV exposure was also significantly associated with low birth weight (aOR 1.91; 95% CI:1.33–2.76, 4 studies, I^2^ = 13%) [41].

### 3.5. Intrauterine Fetal Death

The International Classification of Diseases defines stillbirths or intrauterine fetal death (IUFD) as the death of a fetus that has reached a birth weight of 500 g, or if birth weight is unavailable, gestational age of 22 weeks or crown-to-heel length of 25 cm [51]. On the other side, WHO recommends using the higher limit (1000 g/28 weeks/35 cm) of third-trimester stillbirths for international comparisons and reporting [51,80]. The legal requirements for registration of fetal deaths vary between and even within countries. For example, The American College of Obstetricians and Gynecologists (ACOG) in the USA defines fetal deaths as delivery of fetus with no signs of life whose birth weight is of 350 g or more, or if weight is unknown, of 20 completed weeks gestation or more. In Australia, stillbirth is also defined as fetal death (no signs of life), whether antepartum or intrapartum, at ≥20 weeks of gestation or ≥400 g birthweight if gestational age is unknown. The United Kingdom defines stillbirth as fetal death at 24 or more completed weeks of gestation [51]. The reported incidence of stillbirth varies significantly between studies from different countries and depending on the definitions used but generally ranges from 3.1 to 6.2/1000 births or 1 in 160 deliveries [51,80,81].

The causes may be on the side of the mother, the fetus or the function of the fetoplacental unit. While the etiology remains unknown in about a quarter of cases, one prospective study revealed that nearly two-thirds of cases are attributable to placental dysfunction [51,81]. Infections of the amniotic fluid are responsible for 2–15% of IUFDs (formerly predominantly caused by lues or variola, nowadays mainly by group B streptococci, anaerobic fusobacteria and Listeria monocytogenes, but also by some viruses) [82,83].

There are not many papers studying the association between IUFD and HPV infection, also because the diagnosis of IUFD is rare. Only one study [34] and one meta-analysis [41] met the inclusion criteria for this scoping review. The large retrospective population-based Swedish Medical Birth Register study included 1,044,023 women with singleton deliveries from 1999 to 2016. Compared to the reference group with an intrauterine fetal death of 0.2%, the risk of intrauterine fetal death in the group with HPV positivity detected by cytology was increased to 0.4% (aOR 1.55, 95% CI 1.13–2.12, *p* = 0.006) [34]. Niyibizi et al. included in his meta-analysis only two studies [30,84] meeting the criteria and calculated approximately twice the odds of intrauterine fetal death with proven HPV infection (aOR 2.23; 95% CI: 1.14–4.37, I^2^ = 0.0%) [41].

Slatter et al. observed a potential association between placental HPV infection and intrauterine fetal death. In their study, they declared that 81.2% (13/16) of fetal deaths were diagnosed in placental HPV-positive mothers with no other alteration in health status. They correlated placental HPV infection with a higher risk of fetal deaths similar to other infectious villitis [30]. Subramaniam found that 3.7% (9/242) of pregnant women with a diagnosis of IUFD had abnormal cancer screening results (abnormal cytology or HPV positivity), and only 1.4% (29/2079) had normal results (an assumption of HPV negativity) (aOR 2.6; 95% CI: 1.2–5.8; *p* = 0.01). Because monitoring the frequency of IUFD was not the primary objective of the study, the authors could not definitively establish a firm conclusion regarding this association [84].

## 4. Discussion

Certain infectious pathogens have been repeatedly shown to have a negative effect on pregnancy with severe damage to the newborn. While a causal association between preterm birth and active infection with certain microorganisms (*Ureaplasma urealyticum*, *Mycoplasma hominis*, *Fusobacterium* spp. and *Streptococcus agalactiae*) has been repeatedly confirmed [85,86], evidence for a trigger mechanism for viral infections in preterm birth is limited. One of the reasons is the limited ability to detect specific markers of viral infections [53]. Intrauterine infection activates the immune system, cytokines are secreted and, subsequently, prostaglandins are synthesized, which cause uterine contractions. HPV infection of the vagina or cervix may trigger an inflammatory immune response by altering the vaginal microflora, thereby potentially inducing preterm labour. HPV also infects rapidly replicating trophoblasts and, due to distress of the fetoplacental unit, the tendency to preterm labour is greatly enhanced [46,47,48,49].

HPV disrupts cell cycle regulation, inhibits placental trophoblast growth, reduces their viability and induces cell death [47,87,88]. Human trophoblasts are permissive for HPV, and they have receptors for HPV; thus, they facilitate virus entry into the cell and virus replication [12,30,47]. In vitro studies have shown that the complete HPV life cycle can take place in the trophoblast cell [47]. You et al. also showed that active HPV infection, in addition to reducing the number of trophoblasts, also reduces their ability to adhere to decidua cells [89]. Boulenouar et al. evaluated the response of trophoblastic BeWo cell lines to viral oncogenes E5, E6 and E7 of HPV 16 genotype and observed a significantly reduced growth and adhesion capacity of these cells [90]. This is probably due to the ability of oncoproteins E5, E6 and E7 to inhibit the expression of E-cadherin, a molecule essential for cell adhesion. Expression of viral oncoprotein E5 probably creates hydrophilic pores in cell membranes, leading to osmosis and thus rapid cell death (apoptosis) [47,90]. The influence of viral oncoproteins E6 and E7 results in weakened endometrial cell attachment with embryo expulsion [12,30,88,90]. HPV selectively infects villous trophoblasts and thereby induces spontaneous abortion. Disrupted invasion of extravillous trophoblasts in turn leads to placental dysfunction and preterm birth [12,68,88]. 

Most studies on pregnant women have studied HPV infection in the cervix [91], but HPV infection has also been detected in placenta, amniotic fluid, cord blood, and also in saliva or other oral secretions of newborns and on the surface of fetal membranes, confirming the possibility of vertical transmission of HPV from mother to unborn fetus [46,47,49,92,93,94]. HPV infection of villous and extravillous trophoblasts is likely a contributing factor to adverse pregnancy outcomes. There appears to be a strong correlation between cervicovaginal or urinary HPV infection and placental HPV infection in pregnant women. In one study, 33% (69/207) of women with placental HPV had a positive HPV smear result before pregnancy, compared to 9.4% (8/85) of women with HPV-negative placentae (*p* = 0.0001) [30]. Ambühl et al. observed that only a history of any HPV disease (*p* = 0.032) and cervical cancer history (*p* < 0.001) significantly impacted the presence of placental HPV infection, as opposed to other sociodemographic and clinically relevant parameters [12]. The risk of HPV-positive chorionic villi was found to be four times higher (RR 4.4, 95% CI: 1.6–12.1, *p* = 0.0018) among women with HPV-positive cervical smears who experienced miscarriages compared to those with HPV cervical negativity [15]. A recent study found that midgestational high-risk HPV in urine was significantly associated with placental HPV infection compared to women without high-risk HPV (aOR 13.1, 95% CI: 3.53–73.21; *p* < 0.001) [32]. However, despite a high HPV prevalence in urine samples (40%) among pregnant women and the persistence of infections through to delivery in 52% of them, no significant associations were observed with the investigated pregnancy outcomes [37]. Nevertheless, it is important to note that not all published studies on the potential risk of HPV infection on the course of pregnancy and fetoplacental unit function report the same results. However, individual studies differ significantly in methodology, parameters and size of the study population. For example, when calculating the risk of intrauterine fetal death from a large cohort of 400,583 primiparous women, the authors reached different results when HPV infection from the cervix was diagnosed by cytology (aOR 1.55; 95% CI: 1.13–2.12, *p* = 0.006) or by PCR (aOR 0.93; 95% CI: 0.41–2.09, *p* = 0.86) [34]. Therefore, a definitive conclusion cannot be drawn based on individual studies, but the results of meta-analyses support HPV infection as a risk factor for preterm amniotic fluid leakage and preterm birth (Table 2).

Given the high population prevalence of HPV infection, it is reasonable to assume that HPV vaccination should also improve perinatal outcomes. This assumption is supported, for example, by the findings of a meta-analysis of six studies with 11,869 subjects, which found more than double the risk of female sterility in the presence of HR HPV infection alone (OR 2.33; 95% CI: 1.42–3.83, *p* = 0.0008) [95]. A thorough analysis of health registries from Australia from 2000 to 2015 (a routine vaccination started in Australia in 2007) revealed modest improvements in some perinatal outcomes for mothers who were vaccinated against HPV at a young age. The authors observed a modest decrease in the rate of preterm birth by 3.2% (95% CI: 1.1–5.3) and fewer hypotrophic babies by 9.8% (95% CI: 8.2–11.4) among those vaccinated (after adjusting for other risk factors) [96]. Also, a Finnish study analyzing 20,513 births from a national registry (Finnish Population Register Centre) found that the rate of preterm births was significantly lower in the group of primigravidae previously vaccinated against HPV [97]. Preterm delivery before 37 weeks of gestation was observed in 13/409 (3.2%) primigravidae vaccinated with bivalent HPV vaccine and in 98/1923 (5.1%) primigravidae who did not receive HPV vaccine (OR 0.61, 95% CI: 0.34–1.09). Very preterm delivery before 32 weeks of gestation was not observed in any primigravida vaccinated against HPV (0/409) and in 1.0% (20/1923) primigravidas not vaccinated against HPV (*p* = 0.04) [97].

A major challenge in interpreting the available evidence is the substantial methodological heterogeneity among the included studies. This heterogeneity arises from several key sources. First, HPV detection methods differed considerably. While most studies used PCR-based techniques, others relied on hybrid capture assays or cytology-based classification as a surrogate for HPV infection. The diagnostic sensitivity and specificity of these methods vary, potentially leading to misclassification bias and inconsistent prevalence estimates. Second, the anatomical site of HPV detection was not uniform across studies. HPV was assessed in cervical samples, placental tissue, trophoblasts, urine, and occasionally other biological materials. Given that placental infection may represent a different biological process than transient cervicovaginal colonization, these differences may partly explain discrepancies in reported associations with adverse pregnancy outcomes. Third, not all studies differentiated between low-risk and high-risk HPV genotypes, and only a subset performed genotype-specific analyses (e.g., HPV 16/18). Since oncogenic genotypes may have different biological effects on trophoblastic function, inconsistent genotype stratification further complicates direct comparison between studies. Fourth, the timing of HPV testing during pregnancy varied substantially, ranging from the first trimester to mid-gestation or delivery. Considering that HPV infection may be transient or persistent, differences in sampling time may influence both detection rates and the observed association with pregnancy outcomes. This methodological heterogeneity was most evident in the interpretation of findings concerning the association between HPV infection and miscarriage. One important source of variability was study design. Case–control studies and smaller cohort studies more frequently reported statistically significant associations, whereas larger population-based cohorts often demonstrated weaker or non-significant effects. Smaller studies may be more susceptible to random variation and selection bias, particularly when miscarriage cases are recruited from specialized clinical settings.

Finally, definitions of adverse pregnancy outcomes (e.g., miscarriage, preterm birth, intrauterine fetal death, fetal growth restriction) were not uniform across studies and sometimes followed different national or institutional criteria. Such variability limits the ability to synthesize results and may contribute to the conflicting findings reported in the literature. Taken together, these methodological inconsistencies reduce the comparability of individual studies and must be considered when interpreting the overall body of evidence.

It is also important to emphasize that the evidence summarized in this review is derived predominantly from observational studies. While several associations between HPV infection and adverse pregnancy outcomes have been reported, observational study designs do not permit definitive causal inference. Therefore, the findings should be interpreted as indicative of potential associations rather than proof of causality. Future research should aim to implement standardized HPV detection protocols, consistent genotype stratification, clearly defined pregnancy outcomes, and uniform timing of assessment in order to enhance interpretability and enable more robust synthesis of findings. Furthermore, establishing a causal relationship would require well-designed prospective studies with standardized exposure assessment and adequate control of potential confounding factors. Our scoping review has some limitations. The studies included in this review were heterogeneous in design, and their results cannot be directly interpreted collectively. Some studies combined women with HPV infection confirmed by PCR or hybrid capture assay with those classified as HPV-positive based on cytology results or clinical manifestations used as surrogate markers. Several studies showed already lower adverse pregnancy outcomes in women vaccinated against HPV infection before their sexual debut. Another limitation of this scoping review is that the study protocol was not prospectively registered (e.g., in PROSPERO). Prior registration would have further enhanced transparency and minimized the potential risk of reporting bias. Nevertheless, this review was conducted in accordance with the PRISMA-ScR guidelines, and the methodology was established before the initiation of data extraction. Therefore, this scoping review provides a comprehensive summary of the current knowledge on the investigated topic.

## 5. Conclusions

The adverse effects of some infections on pregnancy and unborn fetuses are well known. Active human papillomavirus infection during pregnancy is likely to negatively affect the health of both mother and child and to increase the risk of specific pregnancy complications, particularly premature rupture of membranes and preterm birth. However, definite conclusions regarding the causal association between HPV infection and other potential adverse pregnancy outcomes cannot be established at this time. Prospective well-designed studies that consider the higher prevalence of HPV infection during pregnancy, as well as all possible pregnancy complications and associated risks, will be necessary to draw more definitive and clear conclusions. The recently demonstrated reduction in pregnancy complications among HPV-vaccinated populations, as shown by several studies, suggests that HPV infection may act as a cofactor in certain adverse perinatal events.

## Figures and Tables

**Table 1:** Search strategy.

Items	Search strategy
Database	Medline PubMed, EMBASE, Global Health, Cochrane Library
Date	Evidence published from 1 January 2000–31 August 2025
Language filter	English only
Spatial filter/Population	No restrictions on region or population
Study type	No restrictions on study design (including systematic reviews and meta-analyses) but studies must have included outcome data
Keywords	(“human papillomavirus” OR “HPV”) AND (“pregnancy” OR “miscarriage” OR “spontaneous abortion” OR “pregnancy loss” OR “preterm birth” OR “premature rupture of membranes” OR “PROM” OR “preeclampsia” OR “eclampsia” OR “pregnancy-induced hypertension” OR “PIHD” OR “fetal growth restriction” OR “FGR” OR “intrauterine growth restriction” OR “IURG” OR “small for gestational age” OR “SGA” OR “intrauterine fetal death” OR “stillbirth” OR “adverse birth outcome”)
Inclusion criteria	HPV infection proven by test polymerase chain reaction (PCR) or hybrid capture assay (HCA) (not valid for meta-analyses) Studies with ≥100 subjects Detailed description of study population Full-text articles reported in English language with clear results of some followed pregnancy outcomes Meta-analyses Articles published from 1 January 2000 to 31 August 2025
Exclusion criteria	Papers without providing clear outcome data Studies with <100 subjects Surrogate for HPV detection (HPV positivity based on cytology results or clinical manifestations, not valid for meta-analyses) HIV positivity of subjects Review articles without calculated statistical data Studies focusing on case reports, abstracts, commentaries Conference papers Studies conducted in a language apart from English

**Table 2:** Characteristics of included studies.

First Author Year Location	Aim of the Study (The Association of HPV Infection)	Study Design	Sample Size HPV Detection Site	Outcome	Key Finding and Discussion
Gomez et al., 2008 [19] PA, USA	1. spontaneous preterm birth 2. preeclampsia	retrospective observational case–control study	108 samples Extravillous trophoblast HPV	Risk of Spontaneous preterm births *p* = 0.03 Preeclampsia *p* = 0.71	Overall DNA HPV prevalence 26.9% (29/108) Rates of apoptosis were 3- to 6-fold greater in transfected cells than in non-transfected cells
Mammas et al., 2010 [24] Greece	1. preterm birth	retrospective observational cohort study	276 women after birth Cervical HR HPV	Preterm birth was significantly associated with HR HPV 16/18 infection, *p* = 0.011	Overall prevalence 53.3% (147/276) Most detected genotypes: HPV 16 (38.8%), HPV 18 (27.9%), HPV 33 (11.6%) The duration of neonatal gestation was significantly lower in HPV-positive mothers (37 weeks vs. 39 weeks, *p* = 0.011)
Bennani et al., 2011 [13] Morocco	1. spontaneous abortion 2. some gynecological pathologies	observational cross-sectional study	751 women Cervical HPV DNA test	Risk of spontaneous abortion aOR 3.76 (95%CI: 1.77–7.98), *p* = 0.001	HPV prevalence 42.5% (319/751) Past history of fibroma, polyp, cyst was risk for spontaneous abortion, aOR 1.686 (95%CI: 1.071–2.654), *p* = 0.026
Zuo et al., 2011 [35] AL, USA	1. preterm birth	retrospective observational cohort study	387 samples placental tissue HPV	Risk of preterm birth *p* < 0.001 (29.4% vs. 10.1%)	Overall placental HPV prevalence 18.1%
Skoczyński et al., 2011 [29] Poland	1. spontaneous abortion (miscarriage)	observational cross-sectional study	129 women (51 with miscarriages and 78 in control group with births) Trofoblast tissue HPV	Risk of spontaneous abortion in HPV positive *p* = 0.366 (24.4% vs. 17.7%) in HPV 16/18 positive *p* = 0.859 (12.8% vs. 11.8%)	This study did not confirm an effect of HPV on the risk of miscarriage, but the prevalence of HPV was one-third higher in women with miscarriages
Cho et al., 2013 [17] South Korea	1. preterm birth 2. PPROM 3. preeclampsia	observational cross-sectional study	311 females after births Cervical HR HPV	Risk of Preterm birth *p* = 0.719 PPROM *p* = 0.029 (aOR 2.32; 95% CI 1.08–4.98) preeclampsia *p* = 0.054	Overall HR HPV prevalence 14.1% Only PPROM was significantly associated with HR HPV infection, no other significant risk factor for PPROM was observed
Conde-Ferráez et al., 2013 [18] Mexico	1. spontaneous abortion (miscarriage)	prospective observational case–control study	281 females (143 cases and 138 controls) Cervical HPV	Risk of Spontaneous abortion OR = 1.80 (95%CI: 0.95–3.45), *p* = 0.0538 No statistical significant association	The most significant risk factors for spontaneous abortion History of >1 abortions OR = 8.67 (95%CI: 2.90–34.75), *p* < 0.001 Age >35 years OR = 8.52 (95%CI: 2.84–34.14), *p* < 0.001 HPV 16 and 58 were the most frequently detected genotypes
Ticconi el al., 2013 [31] Italy	1. recurrent miscarriage	retrospective observational case–control study	524 females (49 cases and 475 controls) Cervical HPV	Significant risk of recurrent miscarriage (HPV prevalence 61.9% vs. 26.5%), *p* < 0.001,	Age range (30–39 years) was the most significant risk factor (*p* < 0.0005) There were no differences in detected HPV genotypes, different method of HPV detection
McDonnold et al., 2014 [25] TX, USA	1. preterm birth 2. preeclampsia	retrospective observational cohort study	314 women with HR-HPV positivity were matched with 628 women with normal PAP smears Cervical HPV	Risk of Spontaneous preterm birth < 35th week OR = 6.85 (95%CI: 1.87–25.09), *p* < 0.001 Preeclampsia OR = 2.18 (95%CI: 1.31–3.65), *p* = 0.004, (10.19% vs. 4.94%) Severe pre-eclampsia OR = 1.93 (95%CI: 0.96–3.87) *p* = 0.09	HPV status based on cytology in two-thirds of cohort Rate of gestational hypertension was lower in the HR-HPV group (2.9% vs. 3.5%). Risk of FGR not significant *p* = 0.53
Slatter et al., 2015 [30] New Zealand	1. preterm birth 2. preeclampsia 3. gestational diabetes mellitus	observational cross-sectional study	339 samples (251 term pregnancies and 88 preterm births) Placental tissue HPV	Risk of Preterm birth OR = 2.13 (95%CI: 1.1–4.0), *p* = 0.018 Preeclampsia and diabetes cases OR = 8.4 (95%CI: 1.9 –51.1), *p* < 0.05	Placental HPV prevalence 75% (253/339) HR HPV prevalence 58% (197/339) Placental HPV prevalence: -preterm birth 84% (74/88) -preeclampsia 100% (20/20) -FGR 76% (55/72) -intrauterine fetal death 81% (13/16) -diabetes 95% (42/44) Cervical HPV prevalence 71% (241/339) Cervical HPV infection was correlated with placental HPV results
Ambühl et al., 2016 [39] Europe, Japan, Mexico, Korea, USA	1. spontaneous preterm birth 2. spontaneous abortion	systematic review, quantitative analyses	14,470 pregnant women (45 studies) Cervical HPV Placental tissue HPV	Cervical HPV prevalence in normal pregnancies 17.5% (95%CI:17.3–17.7) Cervical HPV prevalence in preterm births 47% (95% CI; 42.3–51.6), varied between 15.6% and 67.1%; *p* < 0.0001 Cervical HPV prevalence in spontaneous abortions 24.5%, *p* < 0.05 Placental HPV prevalence in spontaneous abortions 24.9% (95%CI; 22.4–27.5), however varied between 0% and 70.4%; *p* < 0.05	Studies very heterogeneous Full-term pregnancies: -cervical HPV prevalence 17.5% (95% CI; 17.3–17.7), varied 2.2–75% -placental HPV prevalence 8.3% (95% CI; 7.6–9.1), varied 0–47.2% -umbilical cord blood HPV positivity 10.9% (95% CI; 10.1–11.7), varied 0–57.9% Placental tissue of preterm births was only investigated in one study (HPV prevalence 50%) HPV prevalence varied according to the geographical region
Mosbah et al., 2017 [26] Egypt	1. preterm birth	prospsective observational case–control study	103 subjects (53 with preterm births and 50 with term birth) Placental tissue HPV	Risk of Preterm birth *p* = 0.019	Small amount of subjects HPV infection in women with preterm births was statistically significantly higher (18.1% vs. 4.0%)
Ambühl et al., 2017 [12] Denmark	1. preterm birth 2. miscarriage	prospective observational case–control study	271 subjects (103 full-term births, 68 preterm births, 54elective abortions, 46 miscarriages) Placental tissue HPV	Overall placental HPV prevalence 11.4% (31/271) Risk of Miscarriage *p* = 0.63 (10.9% vs. 20.4%) Preterm birth *p* = 0.16 (8.8% vs. 8.7%)	No significant differences Placental HPV prevalence 8.7% in full-term deliveries 8.8% in preterm deliveries 10.9% in miscarriages 20.4% in elective abortions
Hornychova et al., 2018 [20] Czechia	1. preterm prelabour rupture of membranes (PPROM)	prospective observational cohort study	100 women with singleton pregnancies complicated by PPROM Cervical HPV	Prevalence in women with PPROM 24% The rates of intra-amniotic inflammation (21% vs. 18%, *p* = 0.77) and microbial invasion of the amniotic cavity (21% vs. 22%, *p* = 1.00) were not different between groups with and without PPROM	Only women with PPROM in the study HPV cervical infection complicates about one-fourth of PPROM pregnancies, but cervical HPV is not related to a higher risk of inflammatory complications
Caballero et al., 2019 [16] VA, USA	1. preterm birth 2. PPROM	retrospective observational cohort study	612 women with HPV test and 1541 women with cervical cytology results Cervical HPV	Risk of Preterm birth aOR 1.35 (95%CI; 0.89–2.04), *p* = 0.16 PPROM OR 2.07 (95%CI; 1.03–4.14), *p* = 0.04	Overall estimated HPV prevalence 39.5%, but 126 (15.20%) women positive based on HPV test and 703 (84.80%) women based on cervical cytology Maternal HPV infection was associated with an increased risk of PPROM
Aldhous et al., 2019 [11] Scotland	1. preterm birth	retrospective population-based observational cohort study using data linkage	5598 women Cervical HR HPV from Scottish HPV Archive between 1999 and 2015	Risk of preterm birth aOR 1.260 (95%CI; 0.985–1.612), *p* = 0.066	Severe cervical precancerous was significantly associated with risk of preterm birth; aOR 1.843 (95%CI: 1.1 01–3.083), *p* = 0.020
Bober et al., 2019 [14] Poland	1. miscarriage	prospective observational cohort study	143 pregnant women Cervical HPV Trophoblast HPV	The miscarriage was associated with cervical HR HPV (*p* = 0.03) and placental (in trophoblast) HR HPV (*p* = 0.02)	Overall HPV prevalence 13% (19/143); 18% (15/84) in the study group and 7% (4/59) in the control group Similar statistical result for cervical and trophoblast HR HPV positivity
Jaworek et al., 2019 [21] Czechia	1. miscarriage in infertile women	observational cross-sectional laboratory-based study	207 oocyte donors and 945 infertile women (106 pregnancies) Cervical HPV	The risk of miscarriage after IVF + ET aOR 0.95 (95%CI: 0.76,1.18), *p* = 0.616	Overall HPV prevalence 20.3% (234/1152) HR-HPV prevalence was significantly higher in oocyte donors than in infertile women (28.0% vs.16.1%, *p* < 0.001).
Pandey et al., 2019 [28] India	1. miscarriage 2. preterm birth 3. PPROM 4. preeclampsia 5. FGR	prospective observational case–control study	104 pregnant women vaginal HPV in the first trimester	HPV prevalence (cases vs. controls) Miscarriage (4.8% vs. 4.7%) *p* = 0.100 Preeclampsia (7.3% vs. 6.3%) *p* = 0.470 FGR (4.8% vs. 4.7%) *p* = 0.100 PPROM (14.6% vs. 3.2%) *p* = 0.026 Preterm birth (7.3% vs. 3.2%) *p* = 0.324	Overall HPV prevalence 39.4% (41/104) Small size of study population Only the risk of PPROM was significantly associated with the presence of HPV
Kaur et al., 2019 [22] USA	1. preterm birth 2. PPROM 3. preeclampsia 4. FGR	population-based observational cross-sectional study	26,085 pregnant women self-reported vaginal HPV test from Pregnancy Risk Assessment and Monitoring System (PRAMS) between 2004 and 2011	Risk of Preterm birth aOR 0.53 (95%CI 0.29–0.94) PPROM aOR 1.47 (95% CI 0.53–4.06) FGR aOR 1.93 (95% 1.14–3.30) Preeclampsia aOR 0.57 (95% 0.19–1.68)	Overall HPV prevalence only 1.4%, but the true prevalence of HPV could be underestimated due to the self-reported HPV exposure status
Wiik et al., 2021 [34] Sweden	1. preterm birth 2. PPROM 3. intrauterine fetal death	nationwide register-based retrospective observational cohort study	2550 primiparous HPV positive and 11,727 with abnormal cytology compared to 338,109 primiparous with normal cytology as reference group Cervical HPV from Swedish health and population registers between 1999 and 2016	Risk of Preterm delivery aOR 1.19 (95%CI 1.01–1.42); *p* = 0.042 PPROM aOR 1.52 (95% CI 1.18–1.96); *p* < 0.001 Intrauterine death aOR 1.55 (95% 1.13–2.12); *p* = 0.006	The largest observation study The subjects in reference group without HPV test
Niyibizi et al., 2021 [27] Canada	1. preterm birth	prospective multicentric observational cohort study (the HERITAGE study)	899 pregnant women Vaginal HPV (the first and third trimesters) placental tissue HPV	Risk of preterm birth associated: with any vaginal HPV infection aOR 1.39 (95%CI: 0.79–2.46) with HPV 16/18 vaginal infection aOR 3.72 (95%CI: 1.47–9.39) with placental HPV infection aOR 2.53 (95%CI: 1.06–6.03)	Overall HPV DNA vaginal prevalence only 42.0% (378/899) A total of 68.3% (258/378) of HPV-positive women in the first trimester were also positive in the third trimester; 24.4% (63/258) were infected with a new HPV genotype Overall placental prevalence 11.1% (91/819)
Bruno et al., 2023 [15] Italy	1. miscarriage	prospective observational case–control study	100 women (50 with miscarriages, 50 women in control group) Cervical HPV trophoblast tissue HPV	Cervical HPV 34.0% in each group Placental HPV 26.0% vs. 18.0%, RR 1.50 (95%CI: 0.68–3.3), *p* = 0.300	44.1% HPV positive in cervix were positive in placenta The presence of HPV alone is not enough to cause spontaneous abortion, but a high viral load in early pregnancy may increase the risk of negative outcome
Wiik et al., 2023 [33] Norway, Sweden	1. preterm birth 2. PPROM	prospective multicenter observational cohort study	950 pregnant women Urine HPV (in the 16th–22nd week of gestation) Placental tissue HPV	Risk of spontaneous preterm birth aOR 2.26 (CI95%: 0.79–6.50), *p* = 0.13 PPROM aOR 0.62 (CI95%: 0.07–5.59), *p* = 0.67	HPV prevalence at mid-gestation 40% (24% for HR-HPV) HPV prevalence at delivery 28% (16% for HR-HPV) 52% urine HPV positive women had persistence same HR-HPV genotype HPV infection during pregnancy was not significantly associated with increased risk for preterm birth, PPROM, or chorioamnionitis
Værnesbranden et al., 2024 [32] Norway, Sweden	1. preterm birth	prospective population-based observational cohort study	587 pregnant women 587 placental HPV tests 556 urine HPV tests	No woman with preterm birth had placental HPV infection	Placental HPV 3% (18/587) Urine HPV 38% (214/556) HR HPV in urine was significantly associated with placental HPV infection aOR 13.1 (95%CI 3.53–73.21) No increased risk for adverse pregnancy outcomes in women with placental HPV
Liu et al., 2024 [23] China	1. miscarriage 2. preterm birth 3. PPROM 4. preeclampsia 5. FGR	retrospective observational cohort study	6285 pregnant women Cervical HPV (in the 12th–14th week of gestation) Pregnancy outcomes analysis (171 HR-HPV-positive and 171 HR-HPV-negative pregnant women)	Risk with HR HPV Miscarriage *p* = 0.478 (1.2% vs. 0%) Preterm birth *p* = 0.804 (4.7% vs. 5.3%) PPROM *p* = 0.216 (28.8% vs. 22.8%) Preeclampsia *p* = 1 (7.6% vs. 7.6%) FGR *p* = 0.615 (1.8% vs. 0.6%)	HR-HPV prevalence 11.73% the most common HPV 52 (2.90%), HPV 58 (2%), HPV 16 (1.94%), HPV 51 (1.38%), HPV 39 (1.29%). No significant differences between HR HPV-positive and HR HPV-negative groups in the maternal–fetal pregnancy outcomes
Værnesbranden et al., 2024 [37] Norway, Sweden	1.preeclampsia 2. FGR 3. GDM	prospective mother-child observational cohort study	950 urine HPV tests at mid-gestation (in 753 of 950 subjects’ urine HPV tests also at delivery)	Risk with HPV Preeclampsia aOR = 0.55 (CI95%: 0.32–0.93); *p* = 0.024 GDM aOR = 0.56 (CI95%: 0.27–1.15); *p* = 0.114 Risk with HR HPV FGR aOR = 0.52 (CI95%: 0.26–1.02); *p* = 0.058	HPV prevalence at mid-gestation 40%, HR HPV 24%, most common HPV 16 (6%) No evidence was found linking HPV infection to preeclampsia, GDM, or FGR 52% of mid-gestation HPV infections persisted to delivery Persistent infections showed no significant associations with preeclampsia, GDM, or FGR
Lin el al., 2025 [36] China	1.PPROM 2. hypertension 3. cesarian section 4. FGR 5. GDM 6. placental abruption 7.postpartum hemorrhage	retrospective observational cohort study	7110 pregnant women with HPV testing in the second trimester	Risk with HR HPV PROM aOR = 1.29 (95%CI: 1.08–1.55); *p* = 0.005 FGR aOR = 2.07 (95%CI: 1.21–3.54); *p* = 0.008 Risk with HPV (LR + HR) Hypertension aOR 2.73 (95%CI: 1.44–5.15); *p* = 0.002 Cesarian section aOR = 1.19 (95%CI: 1.05–1.35); *p* = 0.006	HPV prevalence 19.9% the most common HPV 52 (5.2%), HPV 16 (2.3%), HPV 58 (1.9%), HPV 42 (1.8%), HPV 51 (1.7%) No statistically significant impact of HPV on placental abruption, postpartum hemorrhage, GDM
He el al., 2025 [38] China	1. miscarriage 2. preterm birth 3. cesarian section 4. FGR	retrospective observational cohort study	6506 women with a total of 13,752 HPV tests from 2016 to 2021 (1069 HPV-positive and 5437 HPV-negative women)	Risk with HR HPV Miscarriage OR = 2.00 (95%CI: 1.26–3.18); *p* = 0.009 Preterm birth OR = 1.64 (95%CI: 1.19–2.26); *p* = 0.020 Cesarian section OR = 1.34 (95%CI: 1.09–1.65); *p* = 0.015 FGR OR = 1.50 (95%CI: 1.08–2.10); *p* = 0.030	HPV prevalence 16.4% the most common HPV 52 (3.3%), HPV 16 (2.3%), HPV 58 (1.0%), HPV 51 (0.6%), HPV 53 (0.5%) HR HPV infection significantly increases adverse pregnancy outcomes compared to both LR and HPV-negative women

**Table 3:** Results of published meta-analyses on HPV’s effect on pregnancy outcomes.

Author Year of Publication	Miscarriage	Preterm Birth	PPROM	Fetal Growth Restriction	Preeclampsia	Intrauterine Fetal Death
Huang et al. [40] 8 studies, 2014	N/A	OR 2.12; *p* < 0.001 (95% CI 1.51–2.98) 8 studies, I^2^ = 61.0%,	N/A	N/A	N/A	N/A
Xiong et al. [43] 18 studies, 2018	OR 1.40 (95% CI 0.56–3.50) 12 studies, I^2^ = 79.4%	OR 2.84 (95% CI 1.95–4.14) 5 studies, I^2^ = 23.5%	N/A	N/A	N/A	N/A
Niyibizi et al., [41] 36 studies, 2020	aOR 1,14 (95% CI 0.40–3.22) 15 studies, I^2^ = 71.0%	aOR 1.50 (95% CI 1.19–1.88) 22 studies, I^2^ = 68.0%	aOR 1.96 (95% CI 1.11–3.45) 6 studies, I^2^ = 0.0%	aOR 1.17 (95% CI 1.01–1.37) 7 studies, I^2^ = 0.0%	aOR 1.24 (95% CI 0.80–1.92) 10 studies, I^2^ = 54.0%	aOR 2.23 (95% CI 1.14–4.37) 2 studies, I^2^ = 0.0%
Wu et al. [42] 7 studies, 2021	N/A	OR 1.81; *p* = 0.002 (95% CI: 1.25–2.62) 2 studies, I^2^ = 0.0%	OR 1.74; *p* < 0.00001; (95% CI: 1.45–2.10) 7 studies, I^2^ = 47.0%	N/A	N/A	N/A
Kovács el al. [44] 13 studies, 2024	OR: 1.02; *p* = 0.054 (95% CI: 0.16–6.31) 3 studies, I^2^ = 66.0%	OR 1.94; *p* = 0.005 (95% CI: 1.31–2.87) 11 studies, I^2^ = 61.0%	not evaluated statistically (lack of sufficient literature)	N/A	N/A	N/A

## Data Availability

No new data were created or analyzed in this study. Data sharing is not applicable to this article.

## Supplementary Materials

The following supporting information can be downloaded at: https://www.mdpi.com/article/10.3390/diagnostics16040629/s1, Table S1: PRISMA 2020 Checklist_The effect of Human Papillomavirus infection on pregnancy outcomes, Figure S1: Engagement strategy framework.

## Abbreviations

ACOG	American College of Obstetricians and Gynecologists
aOR	Adjusted odds ratio, indicates the probability of exposure to cases in the study population and the control population
CI	Confidence interval, upper and lower limits of statistical indicators
DNA	DeoxyriboNucleic acid
FGR	Fetal growth restriction
GDM	Gestational diabetes mellitus
HCA	Hybrid capture assay
HPV	Human papillomavirus
HR	High-risk
I^2^	Index indicates the level of heterogeneity among studies in meta-analysis (I^2^ ≤ 50%, studies are considered homogeneous)
IARC	International Agency for Research on Cancer
IUFD	Intrauterine fetal death
IUGR	Intrauterine growth restriction
LR	Low-risk
N/A	Not applicable
OR	Odds ratio, representing the ratio of the odds of an outcome occurring in The exposed group compared with the non-exposed group—a numerical value (probability) used in statistical evaluation
PCR	Polymerase chain reaction
PPROM	Preterm premature rupture of membranes (amnion) before the 37th week of pregnancy
RR	Relative risk, ratio of incidence rates in HPV positive and HPV negative cohorts
TORCH	(Toxoplasma gondii, rubeola, cytomegalovirus, herpes simplex viruses type 1 and 2)
TPROM	Term PROM, spontaneous rupture of membranes at term before the onset of uterine activity
WHO	World Health Organisation
