# Supplementary material for: The Effect of Human Papillomavirus Infection on Pregnancy Outcomes: A Scoping Review
Source: Diagnostics (Basel). 2026 Feb 21;16(4):629. doi: 10.3390/diagnostics16040629 (PMC12938902; doi:10.3390/diagnostics16040629)
Supplement: Supplementary file 1 [file diagnostics-16-00629-s001.zip › Table S1. PRISMA 2020 Checklist_The effect of Human Papillomavirus infection on pregnancy outcomes.pdf]

Table S1. PRISMA 2020 Checklist

SECTION	ITEM	PRISMA-ScR CHECKLIST ITEM	REPORTED ON PAGE #
**TITLE**			
Title	1	The effect of Human Papillomavirus infection on pregnancy outcomes: a scoping review	1
**ABSTRACT**			
Structured summary	2	Background: Human papillomavirus (HPV) is the most common sexually transmitted viral infection worldwide. Moreover, the prevalence of HPV infection is twice as high in pregnant women as in non-pregnant individuals. Aim of this review was to examine adverse pregnancy outcomes associated with cervicovaginal or placental HPV infection confirmed by a sensitive molecular method.<br>Methods: We conducted searches on major medical databases including PubMed, EMBASE, Global Health, and the Cochrane Library to identify all studies examining HPV infection during pregnancy. Additionally, other online sources were consulted for relevant studies. Thirty records out of the initial 1,868 identified were included in this review for thematic synthesis the PRISMA protocol was followed.<br>Results: This scoping review included a total of 28 studies, 1 systematic review, and 5 meta-analyses. Active HPV infection appears to significantly increase the risk of preterm premature rupture of membranes and preterm birth, as indicated by findings from published meta-analyses and systematic reviews. Determining the association of HPV infection with certain adverse pregnancy outcomes is challenging due to their frequency (such as miscarriage) or rarity (such as intrauterine fetal death). For conditions like pre-eclampsia and intrauterine fetal growth restriction, the limited number of heterogeneous studies precludes definitive conclusions. Moreover, the causes of these outcomes are typically multifactorial. The presence of HPV in trophoblasts and placental tissue is considered crucial for potential adverse pregnancy outcomes. There appears to be a strong correlation between cervicovaginal or urinary HPV infections and placental HPV infections in pregnant women.<br>Conclusion: Persistent HPV infection seems to elevate the risk of preterm premature rupture of membranes and preterm birth. However, definitive conclusions regarding the causal relationship of HPV with these pregnancy complications cannot yet be established. The changes in frequency of certain perinatal complications in populations of women with high HPV vaccination rates may shed more light on this connection	2
**INTRODUCTION**			
Rationale	3	Human papillomavirus (HPV) is the most common sexually transmitted viral infection worldwide. HPV is a group of viruses that 75-80% of sexually active individuals are infected with at least once in their lifetime. Moreover, the prevalence of HPV infection is twice as high in pregnant women as in non-pregnant individuals. Certain infectious pathogens have been repeatedly shown to have a negative effect on pregnancy with severe damage to the newborn. Although the greatest risk	3-4

St. Michael's
Inspired Care.
Inspiring Science.

SECTION	ITEM	PRISMA-ScR CHECKLIST ITEM	REPORTED ON PAGE #
		of HPV infection lies in the initiation of benign and malignant tumours, HPV infection can also adversely affect the course of pregnancy. The exact consequences and implications of HPV infection in pregnant women have not yet been studied in detail, but there is evidence for mechanisms of adverse effects of HPV infection in pregnancy. The exact impact of cervical, vaginal or placenta HPV infection on infection on the pregnant woman and the unborn child is unknown.	
Objectives	4	This scoping review was conducted in order to systematically map the research evaluating the causal relationship between HPV infection, as detected by PCR or HCA in the lower genital tract or trophoblastic tissue (placenta), and adverse pregnancy outcomes such as miscarriage (spontaneous abortion), preterm birth, premature preterm rupture of membranes (PPROM), pre-eclampsia, fetal growth restriction (FGR), and intrauterine fetal death. The following research questions were formulated to identify any existing gaps in knowledge of this field: 1. Is there a significant and causal relationship between HPV infection, as detected by PCR or HCA in the lower genital tract or trophoblastic tissue (placenta), and adverse pregnancy outcomes? 2. Can we estimate the effect of HPV infection on the risk of miscarriage (spontaneous abortion), preterm birth, premature preterm rupture of membranes (PPROM), pre-eclampsia, fetal growth restriction (FGR), and intrauterine fetal death?	5
**METHODS**			
Protocol and registration	5	We did not use any registred review protocol.	6
Eligibility criteria	6	Peerreviewed journal papers were included in this scoping review if they were: published from 1st January 2000 – 31st August 2025, written in English, meta-analyses or studies ≥ 100 subjects with detailed description of study population and HPV infection was proven by test Polymerase Chain reaction (PCR) or Hybrid Capture assay (HCA) (not valid for meta-analyses). Papers were excluded if they did not provide clear outcome data<br>or they did not fit into the conceptual framework a of a scoping review. Review articles without calculated statistical data, studies with surrogate for HPV detection (HPV positivity based on cytology results or clinical manifestations), studies with HIV positivity of subjects, studies focusing on case reports, conference abstracts, commentaries were also excluded.	Table 1
Information sources*	7	To identify potentially relevant documents, the following bibliographic databases were searched from 21st January 2000 to 31st August 2025: Medline PubMed, EMBASE, Global Health, and the Cochrane Library. The search strategies were drafted by an experienced librarian [Martina Habova] and further refined through team discussion. The final search results were exported into EndNote, and duplicates were removed.	6-7
Search	8	A sensitive literature search strategy was developed to locate published peer-reviewed literature, with search terms	6

SECTION	ITEM	PRISMA-ScR CHECKLIST ITEM	REPORTED ON PAGE #
		combined using Boolean operators. Medline Search Strategy (Literature Search performed: September 5, 2025): (“human papillomavirus” OR “HPV”) AND (“pregnancy” OR “miscarriage” OR “spontaneous abortion” OR “pregnancy loss“ OR “preterm birth” OR “premature rupture of membranes” OR “PROM” OR “preeclampsia” OR “eclampsia” OR “pregnancy-induced hypertension“ OR “PIHD“ OR “fetal growth restriction” OR “FGR” OR “intrauterine growth restriction” OR “IURG” OR “small for gestational age“ OR “SGA“ OR “intrauterine fetal death” OR “stillbirth” OR “adverse birth outcome“). The search was limited to research published in English from January 1, 2000 to August 31, 2025.	
Selection of sources of evidence†	9	Data extraction was performed by one reviewer, with three authors deciding which relevant information to extract from the included articles. This process was continuously discussed in an interactive manner by all authors. We extracted information on publication characteristics, study characteristics, and pregnancy outcomes among HPV-positive individuals from each included article, following the PRISMA Extension for Scoping Reviews (ScR) 2020 guidelines..	7
Data charting process‡	10	A data-charting form was jointly developed by one reviewer to determine which variables to extract. The three reviewers independently charted the data, discussed the results and continuously updated the data-charting form in an iterative process..	7
Data items	11	We abstracted data on article characteristics (the first author, year of publication, country of origin), engagement characteristics (;study design, sample size), results (outcome of the study) and formal assessment (key finding and discussion of the study). We abstracted data on meta-analyses characteristics (the first author, year of publication) and results (particular data for the risk of miscarriage (spontaneous abortion), preterm birth, premature preterm rupture of membranes (PPROM), pre-eclampsia, fetal growth restriction (FGR), and intrauterine fetal death.	Table 2<br>Table 3
Critical appraisal of individual sources of evidence§	12	This step is not relevant to this scoping review.	5-7
Synthesis of results	13	We grouped the studies by the inclusion criteria of this scoping review. Where we identified a systematic review, we counted the number of studies included in the review that potentially met our inclusion criteria and noted how many studies had been missed by our search. The results of the meta-analysis are presented separately.	Table 2<br>Table 3
**RESULTS**			
Selection of sources of evidence	14	Active human papillomavirus infection during pregnancy is likely to adversely affect the health of both mother and child and increase the risk of some pregnancy complications, such as premature preterm rupture of membranes and preterm birth. However, definite conclusions about the causal relationship of HPV infection to the above complications cannot be	Table 2<br>Table 3

SECTION	ITEM	PRISMA-ScR CHECKLIST ITEM	REPORTED ON PAGE #
		established at this time. Detailed all relevant outcomes data are shown in supplementary files (Table 2, Table 3).	
Characteristics of sources of evidence	15	The studies' first author, place of origin, study design, sample size, measures and outcomes, and main findings related to each intervention are presented in Table 2.	Table 2
Critical appraisal within sources of evidence	16	This step is not relevant to this scoping review.	5-7
Results of individual sources of evidence	17	Each included source of evidence, which presents the relevant data that were charted that relate to the review questions and objectives is shown in Table 2 or Table 3.	Table 2<br>Table 3
Synthesis of results	18	The data are shown in Figure 2: Engagement Strategy Framework. See related file.	Figure 2
**DISCUSSION**			
Summary of evidence	19	In this scoping review we identified 25 studies, one systematic review, and four meta-analyses addressing the relationship between HPV infection and adverse pregnancy outcomes published from January 1, 2000 to August 31, 2025. Our findings indicate, that active human papillomavirus infection during pregnancy is likely to adversely affect the health of both mother and child and increase the risk of some pregnancy complications, such as premature leakage of amniotic fluid and preterm birth. However, definite conclusions about the causal relationship of HPV infection to the above complications cannot be established at this time.	22
Limitations	20	Our scoping review has some limitations. The studies included in the review were not consistent with a different designs and the results cannot be simply interpreted together. Some studies combined the results of pregnant women with HPV proven by the Polymerase Chain reaction or Hybrid Capture assay and at the same time HPV positivity based on cytology results or clinical manifestations as a surrogate for HPV detection. Several studies showed already lower adverse pregnancy outcomes in women vaccinated against HPV infection before their sexual debut.	22
Conclusions	21	Active human papillomavirus infection during pregnancy is likely to adversely affect the health of both mother and child and increase the risk of some pregnancy complications, such as premature leakage of amniotic fluid and preterm birth. However, definite conclusions about the causal relationship of HPV infection to the above complications cannot be established at this time. Prospective studies considering the higher prevalence of HPV infection in pregnancy as well as all potential pregnancy complications and risks will be necessary to draw more conclusive and clearer conclusions. However, the reduction of pregnancy complications in HPV-vaccinated populations recently demonstrated by several studies suggests that HPV infection is a cofactor of some perinatal adverse events..	22
**FUNDING**			
Funding	22	Literature search and manuscript preparation were supported by the following projects:	25

SECTION	ITEM	PRISMA-ScR CHECKLIST ITEM	REPORTED ON PAGE #
		1. The project National Institute of virology and bacteriology (Programme EXCELES, ID Project No. LX22NPO5103) - Funded by the European Union - Next Generation EU.<br>2. Grant Nr. NW24-09-00505 supported by Ministry of Health of the Czech Republic, and realized at the Department of Gynecology and Obstetrics, University Hospital Královské Vinohrady and Third Faculty of Medicine, Charles University, Prague<br>3. Cooperatio program, Maternal and Childhood Care No. 207035, Third Faculty of medicine, Charles University, Prague<br>No salaries were paid.	

https://www.prisma-statement.org/scoping
