# Supplementary figures and images for: The Effect of Human Papillomavirus Infection on Pregnancy Outcomes: A Scoping Review
Source: Diagnostics (Basel). 2026 Feb 21;16(4):629. doi: 10.3390/diagnostics16040629 (PMC12938902; doi:10.3390/diagnostics16040629)

Figure S1. Engagement strategy framework

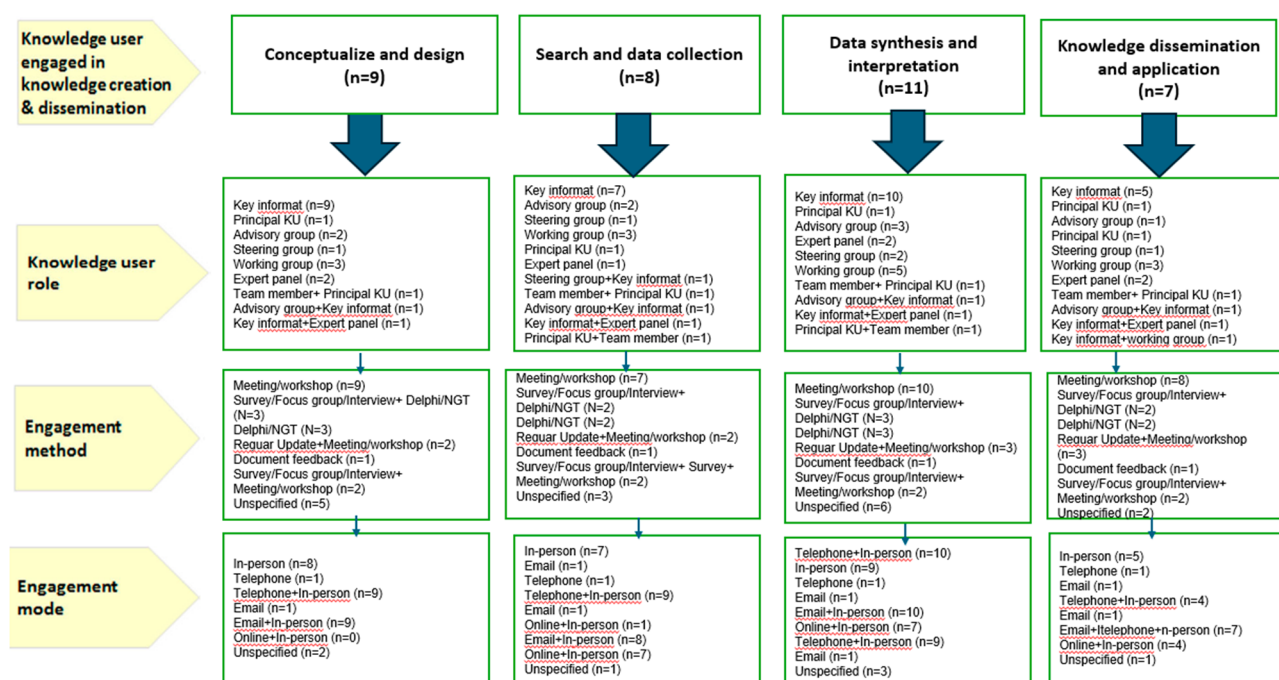

Supplement: Supplementary file 1 [file diagnostics-16-00629-s001.zip › Figure S1. Engagement strategy framework.pdf]
